# Staff motivation and schools' capacities to sustain an intervention to prevent bullying and promote wellbeing in English secondary schools: a qualitative study

**DOI:** 10.3389/fpubh.2025.1559954

**Published:** 2025-04-23

**Authors:** Lauren Herlitz, Chris Bonell

**Affiliations:** ^1^NIHR Children and Families Policy Research Unit, Social Research Institute, Institute of Education - University College London's Faculty of Education and Society, London, United Kingdom; ^2^Department of Public Health, Environments and Society, London School of Hygiene & Tropical Medicine, London, United Kingdom

**Keywords:** sustainment, behavior, whole-school intervention, health, wellbeing

## Abstract

**Introduction:**

Discontinuing effective school health interventions prevents new practices from reaching wider student populations and wastes investment in implementation. While reviews have consistently identified facilitators and barriers to the sustainment of school health interventions, the social processes underlying sustainment remain unclear. We explored the post-trial sustainment of “Learning Together,” a whole-school intervention, found to be effective in preventing bullying and promoting wellbeing in English secondary schools. We examined how staff and students described its sustainment in the 2 years post-trial, what factors staff referred to in explaining their motivation to sustain it, and how schools' capacities affected sustainment.

**Methods and materials:**

Learning Together involved training staff in restorative practice (RP) and supporting schools to implement a staff-student action group and a social and emotional learning curriculum. Using a case-study design, we collected qualitative data from five schools: staff and student interviews 1-year post-trial; staff interviews 2 years post-trial; and descriptive data from the original trial's process evaluation. The General Theory of Implementation guided our thematic analysis.

**Results:**

No school sustained the intervention in its entirety. RP was continued by some individuals in all schools and was sustained at school-level in one school. The curriculum and action groups were discontinued in all schools, although actions initiated by the groups were sustained in two schools. Staff motivation to sustain components was affected by their perceived effectiveness, and individual motivations to sustain RP differed from whole-school commitment to sustaining the approach. Schools' capacities to sustain Learning Together were affected by: the prioritization of academic learning time; the frequent implementation of new initiatives; the timeliness of interventions with school improvements plans; and leadership engagement. Schools needed support to disseminate RP knowledge and skills school-wide and ensure consistent practice, and turnover adversely impacted on knowledge transfer.

**Discussion:**

Sustainment was an intentional, labor-intensive, social process. Intervention developers should consider whether/how interventions are designed to work alongside, replace, or can refine existing practices, and should support schools to mainstream evidence-based interventions to sustain them at school-level.

## Introduction

Schools are a popular setting for child health interventions because of their near universal reach (1–7), with interventions bringing benefits for both health and educational outcomes (8, 9). While health is not schools' core business (10, 11), schools have long been viewed as a setting for health education and promotion, and modeling healthy behaviors and relationships (12–16), while more recently emphasis has been placed on whole-school approaches, particularly to support mental health (17, 18). However, introducing new interventions into schools is costly and places a burden on staff's time, and cognitive and emotional labor (2, 19). Investment is wasted if effective interventions are not sustained (20). Sustaining effective school-based interventions are essential to tackle pervasive health problems such as childhood obesity or poor mental health (21).

Implementation science was developed to address the challenges of embedding new evidence-based interventions into routine practice to improve the quality and effectiveness of health services (22–24), including in school settings (25). Sustaining new practices continues to be one of the largest challenges in translational research (26). It involves organizations investing money, re-organizing staff roles, changing professional norms and taking risks (27, 28). Yet sustainment has been the focus of relatively few studies (29), with synthesis of knowledge hampered by a lack of clarity on the concepts and terminology used (26). Regarding the sustainment of school-based health interventions, a review identified only 24 studies of 18 interventions published from 1996 to 2017 (30). Partial sustainment, where some components were maintained by some schools or staff, was the most common outcome (30). Recent studies have similarly found mixed results (31–34).

Multiple reviews have been carried out on the facilitators of and barriers to intervention sustainment in school settings (30, 35–37). These have consistently identified factors related to: intervention characteristics (e.g., perceived benefits); characteristics of organizations and their local settings (e.g., engagement of school leaders and organizational resources); implementation supports (e.g., training and delivery support); and wider contextual factors (e.g., availability of external funding). However, little research has explored the social processes underlying sustainment (30, 36, 38–40) such as how staff continue to enroll and motivate other colleagues to continue delivery (33) or retain an intervention's profile when new initiatives appear (41). Examining how school staff make sense of interventions and their resultant actions may help us to understand what sustainment strategies may be needed for any school-based intervention, and which may be specific to particular interventions or schools (42).

### Learning Together, a whole-school intervention to prevent bullying and promote health

We examined the sustainment of Learning Together, a whole-school intervention introduced into English secondary schools through a 3-year cluster randomized controlled trial, which ran from 2014 to 2017 in 40 state secondary schools in south-east England, 20 per arm. The intervention aimed to prevent bullying, intentional and repetitive use of physical or psychology force again another individual or group, where there is an imbalance of power between the aggressor and the victim (43). At the start of the trial, nearly a third (32%) of young people reported that they had been bullied at school in the past few months (44). Being a victim of peer bullying is associated with an increased risk of health problems, health risk behaviors such as substance use, and emotional and mental health problems (45) and poorer educational outcomes (46).

Learning Together was found to be effective in reducing bullying, improving students' health and wellbeing, and increasing educational attainment (47–50). The intervention was informed by Markham and Aveyard's (51) theory of human functioning and school organization, which suggests that young people's capacities and goals for healthy or risky behavior are facilitated by increased engagement with education (the school's “instructional order”) and connection to the school community (the school's “regulatory order”) (50). The intervention had three components: (1) restorative practice (RP) involving conversations to prevent or resolve conflicts between students or between staff and students to prevent further harms (52); (2) staff-student action groups in which students and staff collaborate to modify school policies and systems (4, 53); and (3) a social and emotional learning curriculum (54). Table 1 describes Learning Together's components and Supplementary File 1 presents the intervention's theory of change.

**Table 1:** The core components of Learning Together.

Restorative practice (RP)	• In the trial's first year, all school staff received 2–3 h of training in RP approaches, using respectful language to challenge behavior and strengthening relationships. • A further 3-day training was provided for 5–10 staff selected by schools to deliver restorative conferences, more formalized meetings to address instances of bullying, aggression or wrong-doing.
Action groups and locally-decided actions	• Schools formed an “action-group” comprising at least six students and six staff which met six times per year. The group's objectives were to: ○ review data on student health needs and views about the school from an annual student survey (carried out by the trial team); ○ decide local actions to address the needs identified, including how RP was to be used within the school; ○ review and revise relevant school policies to ensure that these supported an inclusive and restorative school environment; and ○ oversee the implementation of the social and emotional learning curriculum. • Schools were asked to recruit diverse students, including those prone to disengagement, and groups had to include a member of the senior leadership team. • For the trial's first 2 years, action groups were supported by a trained external facilitator.
Social and emotional learning curriculum	• Schools were provided with lesson plans and slides to guide teachers' delivery of 5–10 h per year of a social and emotional learning curriculum for students from year groups 8–10 (age 12–15). • The curriculum was designed to complement schools' existing personal, social, health, and economic education provision. Schools were expected to deliver a minimum of 5 h per year.

We purposively selected Learning Together as it was designed to embed into schools' practices through the following sustainment strategies (55, 56): *facilitation*—an external facilitator was recruited in years 1 and 2, with a school staff-member taking on the role in year 3; *promoting adaptability*—schools were encouraged to make local decisions including about how to implement RP and the curriculum and how to improve relationships and student participation (50); *recruiting for leadership*—a senior leader was required to take part in the action group so that the group had the power to implement actions; *changing infrastructure*—schools were asked to review their school rules and policies on behavior to embed the intervention into school procedures.

### Conceptual framework: the General Theory of Implementation

**Figure 1:**
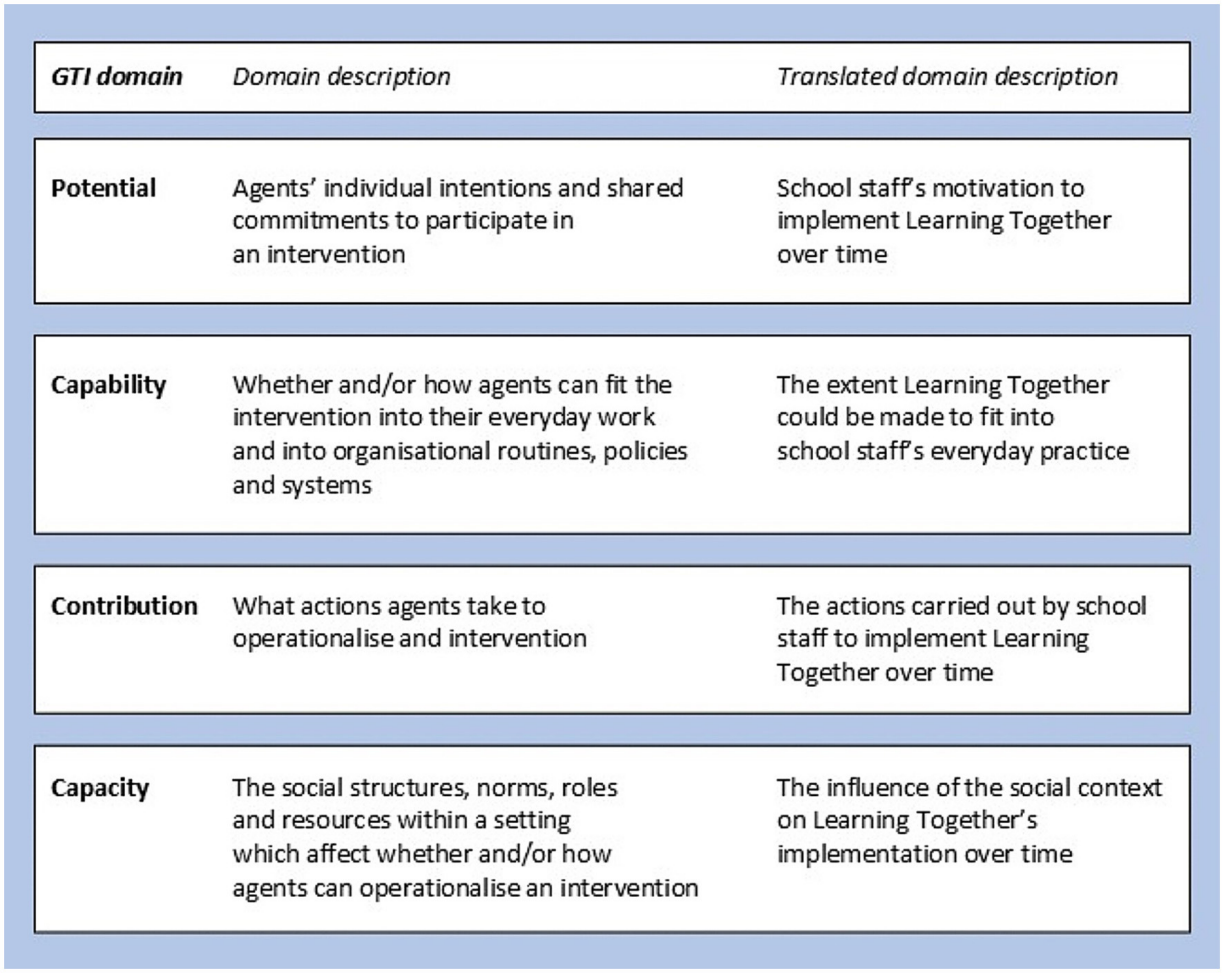
Translation of the General Theory of Implementation (GTI).

We applied the General Theory of Implementation (GTI) to inform our research questions and guide the process of data collection and analysis (57). The GTI is an extension of Normalization Process Theory (NPT), which explains how practices become routinely embedded in everyday life (58) and has been frequently used in healthcare sustainment research (59). The GTI is a sociological framework that examines the dynamic interaction between human agency (people's ability to make things happen through their actions, and the focus of NPT) and dynamic elements of context (the resources that people can draw on to realize that agency) (see Figure 1) (57, 60).

We selected GTI because it focuses on sustainment at an organizational level and enabled us to think about how staff motivations and social norms related to the intervention's objective (in this case, managing students' behavior and health promotion) might affect staff actions. Intervention objectives subject to diverse ideological positions, for example, youth substance use, or relationships and sex education, might pose particular challenges for sustainment (61). While some implementation frameworks consider, at least to some extent, social norms related to intervention objectives under wider sociocultural values/beliefs, societal pressure, and mission alignment (20), or under intervention compatibility (62), it is not well-defined or a central consideration.

The GTI has four key domains, which we translated into terms appropriate to our study (see Figure 1). Supplementary File 2 describes the theory and how it informed data collection and analysis.

This study asks: how did staff and students describe the sustainment of Learning Together in the 2 years following the trial?; what did staff report had affected their motivation to sustain the intervention?; and how did schools' capacities affect intervention sustainment?

## Method and materials

We used a case-study, multi-site study design to explore staff perspectives on sustainment. Case studies enable a rich, in-depth exploration of naturally occurring social phenomenon, and can answer “how” and “why” questions about the processes and meaning of that phenomenon, which can be used to develop or refine theory (63). We adopted a commonly used definition of sustainment: the continuation or discontinuation of intervention activities after the trial had ended, when funding/resources to implement the intervention had stopped (64). The study was approved by the London School of Hygiene & Tropical Medicine Research Ethics Committee (#14223).

### Selection of sites

We selected five schools which were diverse in implementation fidelity in the trial's final year, presuming sustainment would vary among those schools over the next 2 years [see (50) for details on fidelity measurement]. Despite good fidelity in years 1 and 2, in the trial's third year fidelity dropped, particularly for the curriculum (48) (see Supplementary File 3). The 20 schools in the intervention arm were organized into five categories based on the quality of year 3 implementation, ranked from high implementation through to complete discontinuation; one school was selected per category (see Table 2, schools have been pseudonymised). Data were gathered on schools' characteristics. All trial schools had achieved a “good” or “outstanding” rating from Ofsted (the English national school inspectorate). Schools with a “requiring improvement” were excluded from the trial.

**Table 2:** Case study schools' characteristics.

**School**	**Level of implementation during year 3**	**Urban/ suburban**	**Single/ mixed sex**	**Size of student population**	% FSM^*^(past 6 years)
A: Downton Park	Implemented RP^+^, six action groups, and the curriculum.	Suburban	Single	>1,250	10–25%
B: Franklyn	Implemented RP, an adapted form of action groups conducted weekly, and the curriculum.	Urban	Mixed	750–1,000	>50%
C: Fern Grove	Implemented RP and action groups at a lower dose, and discontinued the curriculum.	Urban	Mixed	1,000–1,250	>50%
D: Bletchford	Implemented RP and discontinued the action groups and the curriculum.	Suburban	Mixed	>1,250	25–50%
E: Greenthorne	Discontinued RP, action groups and the curriculum.	Suburban	Mixed	>1,250	< 10%

^+^Restorative practice. ^*^Percentage of students receiving Free School Meals.

### Selection of participants

One year post trial, we purposively sampled school staff and students who had been involved in or continued to deliver the intervention. We contacted the staff-member(s) who had led the intervention during the trial and used snowball sampling to identify other staff. For the three schools that had continued the action groups in year 3, we asked the staff lead to identify two students who might participate. One school (Downton Park) declined to invite any students due to exam pressures. Two years post-trial, we invited the most senior staff interviewed in year 4 to participate. All participants gave written informed consent to take part.

### Data collection

As data collection took place over multiple years, we henceforth refer to the trial years as years 1–3 (school years 2014/15–2016/17), 1-year post-trial as year 4 (2017/18), and two years post-trial as year 5 (2018/19).

Schools sent an opt-out information sheet to students' parents before they were invited to participate. Students interested in participating were given a study information sheet by their teacher and an assent form to sign. At the beginning of each interview, LH checked that students understood the aim of the study, the interview process, and explained confidentiality, re-iterating that participants could choose to not answer a question or stop the interview at any time without having to give a reason. LH checked that students were happy to proceed. Adult interviewees were given an information sheet which was also explained to them in person or by phone and a written consent form to sign. At the beginning of interviews, the aim of the study was described and confidentiality explained. LH reminded school staff that there were no right or wrong answers and that she had no expectations about the sustainment any of the components. All participants gave their written consent to take part.

Staff and students were interviewed between January and March 2018. Interviews aimed to clarify implementation in year 3 (checking details gathered from process evaluation data) and explore whether and how intervention components were delivered and integrated within school systems in year 4. Supplementary File 4 contains the interview guides. If a new initiative started in schools that resembled a component, staff were asked whether they believed it was initiated (fully or partly) because of the intervention or whether it would have happened regardless. LH conducted most staff interviews and all student interviews in-person in a school classroom/office. Several staff interviews were by telephone. Interviews with staff and students lasted around 45 min and 20 min respectively. At least one staff intervention lead was interviewed per school, except for the lead in Bletchford in years 1–2 who declined and one lead at Fern Grove who was on long-term leave.

In year 5, only staff were interviewed. Interviews were conducted in June 2019, focusing on whether and how intervention components were delivered and integrated within school systems in year 5. Trial results on health and behavior outcomes were published in the autumn of year 5 and it was unclear in interviews whether staff were aware of the results (nor were they directly asked). Trial results on educational outcomes were not published during the study period. LH conducted most interviews in-person and two by telephone; interviews lasted around 40 min. In Fern Grove, the identified staff-member had left the school and instead a group interview was conducted with a previous interviewee and another senior leader who had led the action groups and not been interviewed before.

All interviews were audio-recorded and transcribed.

School behavior and anti-bullying policies for year 4 and year 5 were collected from school websites.

### Data analysis

To analyse the components' sustainment, LH conducted a directed content analysis (65) of trial process evaluation data, primary data and policy documents. Interviews were coded deductively to identify: (a) the (dis)continuation of components, (b) the relationship between components and existing practices within the school, and (c) any new activities initiated post-trial which focused on the trial outcomes, i.e., prevent bullying or promoting health. If new initiatives resembled the intervention components, an interpretative assessment was made of whether they aligned with Learning Together's theory of change (see Supplementary File 2).

To examine staff motivation and schools' capacities to sustain the intervention, LH conducted an thematic analysis on the post-trial primary data (66, 67). LH read and re-read interview transcripts to become familiar with the data, and conducted inductive, line-by-line coding using NVivo 12 software. LH checked each code's data for interpretation consistency, recoding as necessary. LH grouped clusters of codes into lower-order themes to summarize patterned response or meaning within the data, with constant comparison used to refine explanations and actively check for examples which did not fit (68). The GTI was used as a sensitizing lens to organize lower-order themes into higher-order organizing themes (see Supplementary File 2). LH discussed the theme development with CB, whether staff's accounts and triangulated with one another and alternative interpretations of staff perspectives. After iterations, they both agreed the final themes. Supplementary File 5 contains a reflexivity statement.

## Results

Interviews were conducted with 18 staff (3 or 4 per school) and 4 students (from two schools) in year 4, and 6 staff (1 or 2 per school) in year 5. Table 3 presents participants' characteristics (participants have been pseudonymised). All staff participants had taken part in the half-day training on RP but not all had participated in the 3-day, in-depth RP training (see Tables 1, 3). All participants had been an action group member for at least 1 year during the trial, except for the senior leader at Bletchford (“Joe”).

**Table 3:** Participants' characteristics.

**School**	**Participant**	Role during the first year post-trial^*^	**Participant attended in-depth training in RP**	**No. of years at the school during the first year post-trial**	**Interviewed during the first year post-trial**	**Interviewed during the second year post-trial**
Downton Park	Angela^+^	Teacher	Yes	>12	✓	
	Callum	Teacher	Yes	< 5	✓	✓
	Victoria	Teacher	Yes	5–8	✓	
Franklyn	Matt^+^	Senior leader	No	5–8	✓	✓
	Gregory	Teacher	Yes	5–8	✓	
	Jessica	Pastoral	Yes	5–8	✓	
	Amelia	Teacher	Yes	>12	✓	
	Craig	Year 11 student	n/a	–	✓	
	Sara	Year 11 student	n/a	–	✓	
Fern Grove	David^+^	Senior leader	Yes	5–8	✓	
	Harriet^+^	Senior leader	No	5–8		✓
	June	Pastoral	No	9–12	✓	✓
	Katie	Teacher	Yes	< 5	✓	
	Harry	Year 10 student	n/a	–	✓	
	Kristen	Year 9 student	n/a	–	✓	
Bletchford	Joe	Senior leader	No	>12	✓	✓
	Brett	Teacher	No	9–12	✓	
	Jenny^+^	Teacher	Yes	9–12	✓	
	Penny	Pastoral	Yes	>12	✓	
Greenthorne	Colin^+^	Senior leader	No	>12	✓	✓
	Amy	Teacher	Yes	9–12	✓	
	Toby	Teacher	Yes	>12	✓	
	Paul	Teacher	Yes	>12	✓	

^+^Intervention lead during the trial. ^*^Year 9 is equivalent to age 13/14, Year 10 to age 14/15, and Year 11 to age 15/16.

### The sustainment of Learning Together

#### Restorative practice (RP)

RP was the most Successfully Sustained Component; *all* staff interviewed continued to use RP in some form in their individual practice in years 4 and 5. However, the degree to which the approach was embedded across each school varied greatly.

#### Staff-student action groups

Staff reported that all five schools had discontinued the original action groups by the end of year 3. Locally decided actions were sustained at Franklyn and Fern Grove into year 5, and both schools created new funded positions to focus on student voice and engagement. Local actions at Greenthorne and Bletchford were not sustained beyond year 2, and at Downton Park, the action groups were primarily used as a forum for deciding how students and staff could learn about RP approaches. Four schools created new staff-student groups that engaged a diversity of students and aligned with Learning Together's theory of change.

#### Social and emotional learning curriculum

The curriculum was the least sustained component. Three schools had already discontinued the curriculum by the end of the trial (Bletchford, Greenthorne and Fern Grove). Franklyn discontinued it in year 4. Staff at Downton Park could not confirm whether the curriculum had been used beyond the trial.

Supplementary File 6 presents a detailed summary of the sustainment of Learning Together in each school.

### Staff motivation and schools' capacities to sustain Learning Together beyond the trial

We identified two themes related to staff's motivation to sustain components and six themes related to schools' capacities to sustain the intervention (see Table 4).

**Table 4:** Summary of themes and their alignment with the GTI.

**Research question**	**Theme**	**Alignment with GTI domain**
Staff motivation to sustain the intervention	The perceived effectiveness of components compared to existing practices	*Contribution*: “coherence,” making sense of the value of a component in relation to existing practices, and “reflexive monitoring,” formally or informally evaluating the effects of a component.
	Differences between staff's individual intentions and whole-school commitment to sustaining RP	*Potential*: “individual intentions” and “shared commitment”
Schools' capacities to sustain the intervention	The prioritization of academic learning time	*Capacity*: “social norms,” rules of membership and participation in an intervention
	Coping with the continual stream of new initiatives in schools	
	The timeliness of the intervention regarding the school's strategic priorities	
	The vital role of senior and middle leaders in sustaining new practices	*Capacity*: “social roles,” expectations of participants in an intervention
	Varied approaches to disseminating knowledge across a school	*Capacity*: ‘cognitive resources', participants' access to knowledge and information needed to operationalise an intervention
	Staff turnover had a significant impact on the transfer of knowledge	

#### The perceived effectiveness of components compared to existing practices

Until year 5, school staff were unaware of the reported effectiveness of the intervention. Nonetheless, staff made judgements about each component's effectiveness based on their own experiences and observations of whether students and/or staff positively engaged with and gained insight from activities, and whether these led to improved student behavior compared with existing practices. These experiences appeared central to staff motivation to sustain practices as individuals and aligned with the GTI dimensions of “reflexive monitoring” and “coherence” under “Contribution”—practitioners interpreting information about an intervention's effects and it contributing to making sense of an intervention.

RP was perceived as effective in improving behavior and relationships in comparison to punitive approaches, while the curriculum was considered to add little value to existing personal, social, health and economic (PSHE) education curricula (see Supplementary File 7 for further theme details). Perceptions of the action groups' effectiveness varied depending on whether it had achieved purposeful actions in its first 2 years, meaningfully engaged a diverse group of students in revising the school's behavior rules or policies, and offered something different to existing student councils:

*The points that we raised [in the action groups], I can't help but feel that they might have been raised through school council and school congress anyway… Paul, staff, Greenthorne, year 4*

Staff at Bletchford and Downton Park said that they had used the groups mainly to consult with students about implementing RP rather than asking students to co-develop actions about behavior management and how to improve staff-student relationships. Even though staff at Fern Grove and Franklyn thought the action groups had been effective in reviewing school behavior policy and co-producing changes to rules, rewards and sanctions, they were not sustained, as staff perceived the student council to be the embedded system for student voice and any local actions required ongoing staff training and monitoring that was beyond its remit.

#### Differences between staff's individual intentions and whole-school commitment to sustaining RP

Staff described beliefs and attitudes about pupil behavior that affected their intentions to use RP approaches. This aligned with the GTI domain of “Potential”—the individual autonomy of practitioners to pursue their interests and collective commitment to organizational change. We did not find evidence of differences in staff beliefs in adopting a staff-student action groups or a curriculum.

Staff-members who were involved with Learning Together tended to have a high level of readiness to adopt RP approaches. Many staff across the five schools reported in year 4 that the RP techniques that they had learnt on the in-depth training labeled and scaffolded their existing practices; “*It just gave me more of a.. name to what I was doing*” (Brett, staff, Bletchford, year 4). However, staff were uncertain of how to change the behavior of colleagues who were resistant to change so that the intervention could be mainstreamed across the school.

*We knew that for some staff… the idea that they might need to sit down in a restorative meeting and then, themselves, apologize, or… reflect on their own behavior… and actually understand their own role in that sort of relationship, was going to be very difficult*. David, senior leader, Fern Grove, year 4

Staff reported that some colleagues felt that discussing behavior with students or accepting some role in how incidents manifested undermined their authority. Staff were wary of being seen to criticize another teacher's handling of a situation and reported that there were no quick solutions to changing their beliefs and attitudes. They also reported that the emotive nature of behavior management could increase resistance to using RP when staff were under stress. Staff highlighted that RP introduced uncertainty into how staff could respond consistently to students' behavior because it required staff discretion. It was difficult for some staff to understand whether RP was a replacement for punishments or whether it could complement detentions or other sanctions:

*Some people thought we'd gone to… restorative ways of dealing with things and then not having… trying not to use detentions as much… Some people were just going on as normal and just using the old system. And some people were kind of…doing a mixture… And maybe… having a restorative discussion with a pupil about something that had happened, but then still issuing a sanction… I think… there definitely wasn't a consistent approach*. Brett, staff, Bletchford, year 4

In contrast, detentions were considered more consistent because any teacher could read the school's behavioral policy and deliver a detention without needing special skills. There was an intuitive understanding of the value of detentions (more detentions and after-school/weekend detentions equalled worse behavior) and they could be measured and monitored easily. Staff had needed to understand how RP could be integrated into schools' discipline systems and policies.

#### The prioritization of academic learning time

Most staff reported that the inflexibility of the school timetable and the lack of time that was available for non-teaching activities increased the difficulty of integrating Learning Together systemically in schools. This aligned with the GTI dimension of “social norms” under “Capacity” – school-sanctioned rules about what constituted “teaching work” and how it was delivered, shaped practitioners' participation in the intervention:

*If we're going to change things seriously, we need to be given time… “Right, let's assess this, let's think it all through. Let's think about how we're going to plan it.” Without worrying about, “I've got to mark those books for tomorrow, I've got to plan those lessons for tomorrow… I've got this trip I'm organizing next week, I've got all that paperwork to get in for next Tuesday.”* Callum, staff, Downton Park, year 4

The lack of “free” time affected staff's ability to carry out RP because it required staff to find time to discuss disciplinary incidents with individual students. In contrast, detentions were considered time-efficient because they were quick to issue, they did not need to be carried out by the teacher who had issued them and could be delivered to multiple students at once. Timetabling also affected commitment to the action groups as it was difficult for the same group of staff and students to meet regularly outside of lesson time. The curriculum was a poor fit with timetabling at Greenthorne, Franklyn and Fern Grove, and staff had to adapt it, giving them unwanted additional work and lowering their motivation to sustain its use.

#### Coping with the continual stream of new initiatives in schools

The continual stream of initiatives in schools affected staff's commitment and ability to systemically integrate interventions, supporting the GTI dimension of “social norms” under “Capacity” because it explained “typical” organizational behavior in relation to new initiatives. Staff explained that schools were constantly participating in new education and health initiatives, whether from policy mandates, their own interventions, or by invitation from local government or other external providers which provide free or subsidized training and resources. Staff reported that it was difficult to maintain the profile of Learning Together within this context. Staff were cynical that new initiatives would be meaningful for their practice, and the stream of new initiatives made it difficult to consolidate learning and commit to sustaining approaches:

*It can be… tricky because you're constantly having to re-familiarize yourself with new ways of doing things. And… really it is at the cost of the student a lot of the time because there's so much time taken for us to invest in getting to grips with… “What do we have to show now? How do we have to show this? What evidence do we need to show?”* Angela, staff, Downton Park, year 4

#### The timeliness of the intervention regarding the school's strategic priorities

Sustainment was affected by whether the intervention aligned with a school's strategic priorities in its school development/improvement plan, aligning with the GTI dimension of ‘social norms' under ‘Capacity' because plans shaped agreed ways of working. Staff reported that if a school intended to review its behavior policies, Learning Together's approaches and activities had a better chance of being given more attention and resources. Once the school deemed behavior a priority, a school's commitment to an intervention was increased:

*Once it's listed as a priority, then sufficient… training time, briefing time, the ability to stand up, the ability to make a fuss and do things, that comes with that*. David, senior leader, Fern Grove, year 4

When Learning Together had aligned with the school's improvement plan, senior leaders at Bletchford had paid for an external consultancy to help them develop behavior policies and provide staff training and coaching. Senior leaders at Fern Grove and Franklyn had found money to support new student voice posts (additional paid responsibilities for existing staff members).

Greenthorne staff noted that behavioral management was not prioritized by the school during the trial or in year 4. However, in year 5, senior leader Colin reported improved conditions for introducing more student consultation and RP approaches after a new head teacher was appointed and a revised Ofsted inspection framework increased focus on students' resilience.

#### The vital role of senior and middle leaders in sustaining new practices

Staff reported that both a committed middle leader and senior leader were crucial for sustaining new practices, supporting the GTI dimension of ‘social roles' under ‘Capacity' which frame staff behaviors to sustain an intervention. An action-focused, hard-working and communicative middle leader could maintain intervention operations, train staff, and encourage staff and students' enthusiasm for initiatives. However, a senior leader was also essential to: change school systems and policies; legitimize the use of RP and authorize a change to discipline procedures and spending on initiatives (e.g., for additional training); encourage staff to buy into a new approach; and monitor and challenge staff on adopting and maintaining new practices.

*We [senior leaders] made it very clear that… you know, trying to be encouraging, to say, “Look, it is difficult; but we all have to try. And although we'll always get it wrong and we won't be perfect, the next time we still… we don't just throw it out and give up.”* Joe, senior leader, Bletchford, year 5

Three schools (Greenthorne, Bletchford, and Downton Park) seconded a middle leader (that is, a head of year or equivalent) to a senior position to meet the trial requirement that a senior leader must be involved in the action group. However, the events that transpired indicated that the seconded leaders did not have the necessary authority to change discipline system; in one school, senior leaders blocked their suggested changes to schools' behavioral policies and rules, and in another, senior leaders changed the policies without consulting with the seconded staff member.

#### Varied approaches to disseminating knowledge across a school

All staff were in some way responsible for students' behavior (see Supplementary File 8). The significance of having distributed behavioral responsibilities was that it required considerable effort, leadership and co-ordination to transfer knowledge and expertise about RP or local actions across the school. In the absence of guidance from trial leaders, staff intervention leads approached this very differently (see Table 5). This aligned with the GTI dimension of “cognitive resources” under “Capacity,” which explains that people's access to knowledge and information affects intervention delivery.

**Table 5:** Schools' approaches used to sustain the dissemination of knowledge about RP and local actions.

**Approach**	**School(s)**
Making a plan for rolling-out RP across the school	Bletchford
Creating a working group to focus on the intervention roll-out	Bletchford
Creating publicity materials	Downton Park; Fern Grove; Franklyn; Greenthorne
One-off inhouse training session for staff	Downton Park; Fern Grove Franklyn; Greenthorne
Multiple training sessions for staff	Bletchford
Training sessions for new staff joining the school	Bletchford; Downton Park
One-off inhouse training session for students	Bletchford; Downton Park
Providing feedback to staff on their use of RP approaches in staff meetings	Fern Grove
One-to-one coaching for staff	Bletchford; Franklyn
RP approach explicitly mentioned in schools' behavior policy	Bletchford; Downton Park; Fern Grove; Franklyn; Greenthorne
Use of external consultancy to embed RP principles in the behavioral system	Bletchford

Staff at Bletchford had a comprehensive approach to sustaining RP knowledge dissemination by creating a working group to support its roll out, offering multiple training sessions to all staff, and investing in support from an external consultancy to help them embed the principles in their behavioral system. Staff at Downton Park also used multiple strategies to disseminate RP, it was not integrated into behavioral policy. The other schools implemented short-term dissemination strategies relating to RP or local actions, e.g., one-off training sessions.

Many staff reported that transferring knowledge and expertise in RP needed to go beyond explaining the principles to demonstrating how it worked in practice through modeling, role play, coaching and observation. Several staff highlighted the importance of adults modeling daily the RP principles which they wanted to see from their students. This implied that sustaining knowledge dissemination in RP required active and extensive work:

“*When you just speak to a member of staff in an hour [for training], and then they go into a lesson – surprise, it all crumbles! So what we're doing is making sure that staff are spending more time observing lessons… finding out… what does it actually mean to embody a restorative approach*.” Matt, senior leader, Franklyn, year 5

#### Staff turnover had a significant impact on the transfer of knowledge

Staff turnover caused significant challenges for retaining intervention knowledge and expertise, supporting the GTI dimension of “cognitive resources” under “Capacity.” Staff interviewed could recall retention information about 32 of the 40 staff who had received in-depth training (with missing data spread across the schools). A third of trained staff (34%, 11/32) had left during the trial, with turnover particularly high for Fern Grove, a large urban school. Staff explained that departing colleagues took intervention knowledge and skills with them and several staff highlighted that they had received no hand over on the intervention's aim and approach when they had taken over from a departing staff member. If new staff were not trained, this stagnated progress on embedding RP within the school. Staff noted that high staff turnover also contributed to overall staff stress level, particularly if roles were left unfilled due to budget constraints.

However, turnover also had potential benefits. Two staff noted that greater progress could be made if staff who were resistant to culture change left. Colin, a senior leader at Greenthorne, reflected that turnover could also give staff with RP knowledge and skills an opportunity to progress. For example, Amy was a deputy head of year when she was trained in RP in year 1. By year 4, she had progressed to head of year and reported that she continued to use RP in her work and, beyond year 5, Colin reported that she would become head of key stage 4 (ages 14 to 16).

## Discussion

### Summary of findings

We examined the sustainment of a whole-school intervention, focusing on the dynamic interaction between the components, staff delivery and the school context. Sustainment was an intentional, labor-intensive, social process, affected by staff beliefs about components' purpose and effectiveness, mediated by accepted ways of working individually and organisationally and avenues for disseminating and embedding knowledge. Intervention benefits needed to be persuasive to make this effort worthwhile, alongside plans to embed practice through training, monitoring, and changes to organizational infrastructure. RP was the most sustained component, maintained in individual practice because of its perceived effectiveness, and at whole-school level in one school where staff planned strategies to diffuse RP knowledge and skills across the school, and senior leaders invested in support from an external consultancy.

While other studies have focused on intervention characteristics (20), this study extends the literature by highlighting how the objective of an intervention shapes sustainment processes. Components were interpreted and appraised in relation to existing practices regarding behavior management and health promotion (detentions and other sanctions, student councils, and PSHE). Staff reported behavior-management responsibilities were distributed across the school and that school-wide consistency in behavior management was vital. A*ll* staff needed training and guidelines that explained how RP could be made to work with existing discipline procedures. Staff viewed behavior management as a skill learnt through active demonstration, modeling and coaching, and facilitated by high staff wellbeing, indicating extensive implementation and sustainment support was needed to support a whole-school approach, particularly for staff with ‘traditional' disciplinarian values (33). Studies suggests that teachers rely on personal experiences rather than on research evidence of effectiveness when deciding on approaches to use (69–71), and other studies of whole-school behavior management interviews have found staff philosophical agreement with intervention principles to be important to sustainment (72).

### Implications for practice

The study highlighted particular aspects of the school setting that affected sustainment, supporting an ecological perspective (73, 74). Staff prioritized academic instructional time which meant there was minimal time for them to engage with individual or small groups of students outside of the classroom or to devote attention to embedding an intervention. Turnover in new initiatives also dampened their motivation to invest in work to integrate the intervention, for example, by building a community of practice or monitoring its implementation and providing feedback on its use. These challenges have been noted in other studies of behavior management interventions (1, 33, 75). This suggests that implementing and sustaining changes to staff behavior management practices requires both additional staff resources and dedicated staff capacity, where staff workloads and responsibilities in relation to initiatives are made explicit (76).

Genuine leadership involvement (that is, not seconded leadership) and revision to school rules and policies were key sustainment strategies highlighted in this study. They legitimized and maintained the intervention's profile and delivery, supporting other evidence on embedding interventions in organizational systems (77, 78). Early conversations with senior leaders about their school vision and improvement priorities could help to identify schools more disposed to embedding an intervention (79, 80). Leadership training and coaching on implementation could further support sustainment, for example, the Implementation Leadership Scale identifies specific leader behaviors that can support implementation, for example, proactively developing plans, removing obstacles to implementation, and setting standards for practice (81).

Funders, practitioners and researchers must recognize that embedding new practices takes time, and there are many threats to the continuation of a programme, including competing priorities, low resources, low staff capacity, and staff turnover (20, 37, 76). By identifying and addressing risks, schools leaders and other stakeholders wishing to sustain a new initiative may be able to mitigate them, for example, by taking into account staff turnover rates when planning staff training and coaching (77, 79, 82). Studies that have examined sustainment strategies in school and healthcare settings have identified additional sustainment strategies which could bring benefits: involving students and families (or patients) in designing intervention content and implementation to catalyze change, investing in workforce training, planning for ongoing promotion of the initiative, embedding measurement and monitoring of implementation progress as proxy measures of success, and bringing in further funding (72, 76, 83). Further research is needed to test to impact of different strategies on sustainment.

### Implications for research

The study shed light on the acceptability and feasibility of the long-term implementation of the intervention. Clarity was needed on whether components were designed to replace or work alongside existing practices, or whether the intervention could have refined existing practices (84). For example, to improve school engagement among disengaged students, would it have been equally effective but more sustainable to work with schools to improve the diversity of existing student councils rather than create separate action groups? Findings support the UK Medical Research Council's framework for developing and evaluating complex interventions, which suggest evaluators should consider how the intervention interacts with its context and how varied stakeholder perspectives can be included in the research (85). Context is broadly defined in the framework, so we suggest developers start by considering what existing practices or policies within the setting have similar goals to the proposed intervention.

Over the last decade, there has been a shift from considering deviations from the original intervention protocol to be an implementation failure (86, 87) to thinking of adaptation as a key part of the sustainment process to improve an intervention's fit or effectiveness in its setting (88, 89). However, evidence from our study suggests that not all adaptations benefitted sustainment. Some adaptations were made in response to contextual problems without regard to the interventions theoretical rationale (90, 91), for example, using the action groups to implement RP rather than co-developing actions and revising policies with students because schools were unwilling to change school policies. Other adaptations such as new action groups focused on social inequalities were sustained. The evidence on how adaptation affects sustainability is weak with most studies failing to describe adaptations or examine their impact on outcomes (37, 60, 92). Further research is needed into how and why adaptations are made, and their impact on sustainment, including the lasting effectiveness of interventions (89, 93, 94).

The GTI was valuable in helping to explain the complexity of the intervention, particularly the interaction between the intervention, practitioners and the setting (74). This theory contrasts with frameworks that set out detailed factors or precursors to sustainment (37, 95), and with the NPT's narrower focus on the operationalisation of complex interventions (60). Use of the GTI helped reveal that sustainment could be conceived as the process of moving the intervention implementation from a small group of enthused individuals to harnessing mainstream organizational support and utilizing organizational policies and systems. Most GTI dimensions that were initially organized under the domains of “capability” or “contribution” could be subsumed under domains of “potential” and “capacity,” suggesting that “potential” and “capacity” were the most valuable domains for examining sustainment processes. As the GTI primarily focuses on practitioners, the GTI may less suited studying interventions which involve the parent community or other stakeholders (96).

### Limitations

We did not examine sustainment outcomes (e.g., sustained positive impacts on bullying and pupil's wellbeing). We relied on participants' reports and did not use independent observation or validation. Only the staff who were closely involved with the intervention were interviewed; the findings do not consider the perspectives of staff and students that chose not to participate it in or who were not involved its delivery. These perspectives could have strengthened findings on mainstreaming the intervention in the school. Participating staff may have experienced social desirability bias, wishing to present themselves, their colleagues or school in a favorable light, for example, overestimating their continued use of RP or downplaying tensions with coworkers. We tried to encourage participants to share their perspectives by emphasizing our neutral stance on the intervention's sustainment and creating an open and comfortable atmosphere in interviews. All participants identified difficulties with sustainment, suggested that selection bias was limited. Most participants appeared to be reflective, articulate and open about their experiences; only two participants interviewed by telephone appeared to be more reluctant to share views that they perceived might disparate their school or colleagues.

Case studies aim to develop analytic generalizations as opposed to statistical generalizations, enabling the findings from one study to be used to develop understanding about the phenomenon in another situation (63). Our findings are based on a small number of schools and a larger sample may have revealed further depth of understanding on the phenomenon of sustaining a whole-school bullying prevention intervention.

The schools that participated all had good or outstanding Ofsted ratings. The findings may be less applicable to sustainment in schools that have lower achievement and/or capacity; these schools may have a higher impetus to change and embrace new interventions or have even less capacity to devote to non-academic activities. Although out of scope for this study, future research could employ mixed-methods to collect quantitative data on sustainment across all schools that implemented the intervention to contextualize the qualitative data. The School-wide Universal Behavior Sustainability Index- School Teams (SUBSIST) measures of sustainability determinants has been rated highly for its psychometric properties and pragmatic use and could be adapted for an English school context (97, 98).

## Conclusion

Sustainment was an intentional, labor-intensive, social process, affected by staff beliefs about components' effectiveness, mediated by accepted ways of working individually and organisationally and avenues for disseminating and embedding knowledge. Intervention developers need to pay greater attention to whether components are designed to work alongside or replace existing practices, or whether they can refine existing practice. Schools need support to mainstream interventions that are evidence-based and perceived as effective by staff. There is a significant gap in our understanding of how to scale-up and sustain interventions and further research is needed to understand the reasons for adaptation and its impact on sustainment.

## Data Availability

The raw data supporting the conclusions of this article will be made available by the authors, without undue reservation.
