# Supplementary figures and images for: Staff motivation and schools' capacities to sustain an intervention to prevent bullying and promote wellbeing in English secondary schools: a qualitative study
Source: Front Public Health. 2025 Apr 23;13:1559954. doi: 10.3389/fpubh.2025.1559954 (PMC12057643; doi:10.3389/fpubh.2025.1559954)

# Supplementary file 1: Learning Together’s theory of change


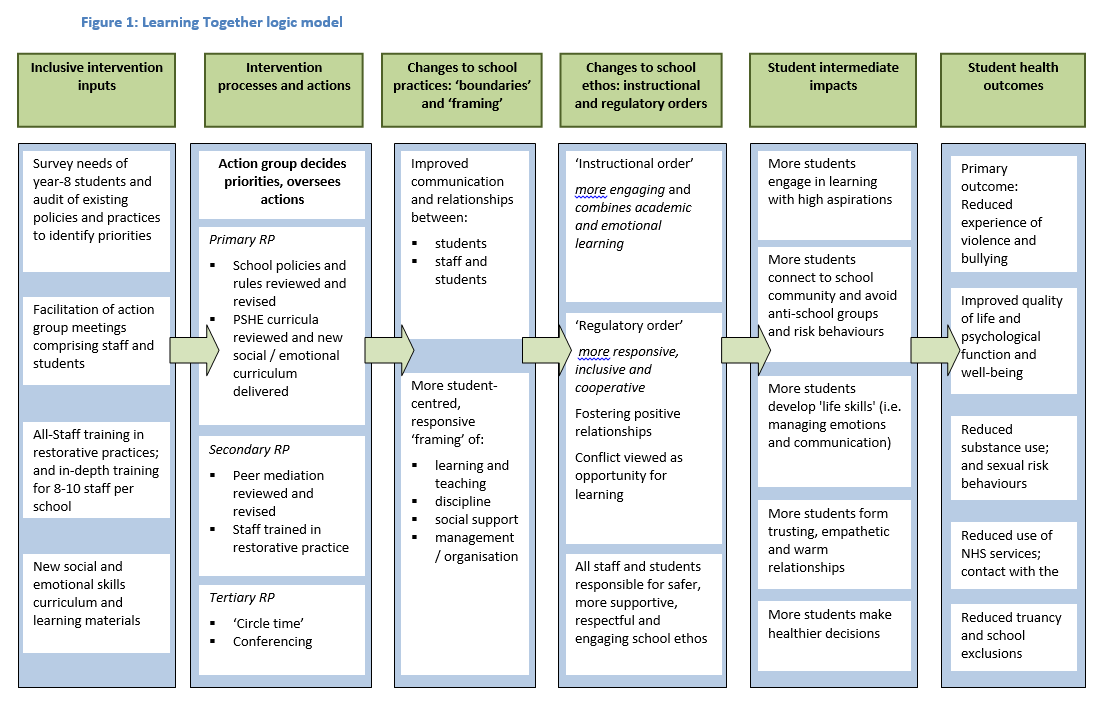

Supplement: Supplementary file 1 [file Supplementary_file_1.docx]
