# Supplementary material for: Staff motivation and schools' capacities to sustain an intervention to prevent bullying and promote wellbeing in English secondary schools: a qualitative study
Source: Front Public Health. 2025 Apr 23;13:1559954. doi: 10.3389/fpubh.2025.1559954 (PMC12057643; doi:10.3389/fpubh.2025.1559954)
Supplement: Supplementary file 2 [file Supplementary_file_2.docx]

# Additional file 2: The General Theory of Implementation and how it informed data collection and analysis

## The General Theory of Implementation (GTI)

The GTI examines the dynamic interaction between human agency (people’s ability to make things happen through their actions, and the focus of NPT) and dynamic elements of context (the resources that people can draw on to realise that agency) (see figure 1) (C. May 2013). People’s actions to sustain an intervention may be mobilised or hindered by social structural resources, such as social roles, social norms and material resources (e.g. funds/equipment/space), or social cognitive resources such as staff’s commitment to learning and implementing a new behavioural approach.

The GTI is comprised of four domains:

- *potential* – the social-cognitive resources available to agents, that is, whether individual agents, and/or the organisations in which they act, have the desire and ability to participate in an intervention.
- *capability* – the possibilities presented by a complex intervention – whether and/or how agents can fit the intervention into their everyday work and into organisational routines, policies and systems;
- *contribution* – what agents do to implement a complex intervention, that is, what actions agents take to operationalise an intervention (see figure 1); and
- *capacity* – the social-structural resources available to agents. These are the social structures, norms, roles and resources within a setting which affect whether and/or how agents can operationalise an intervention.

Figure 1 presents on overview of the theory’s domains and dimensions. The domains are not linear or sequential but are thought to interact continuously with each other in emergent and complex ways (C. May 2013). Each domain and its dimensions are described in the sections that follow.

Figure 1: The general theory of implementation domains
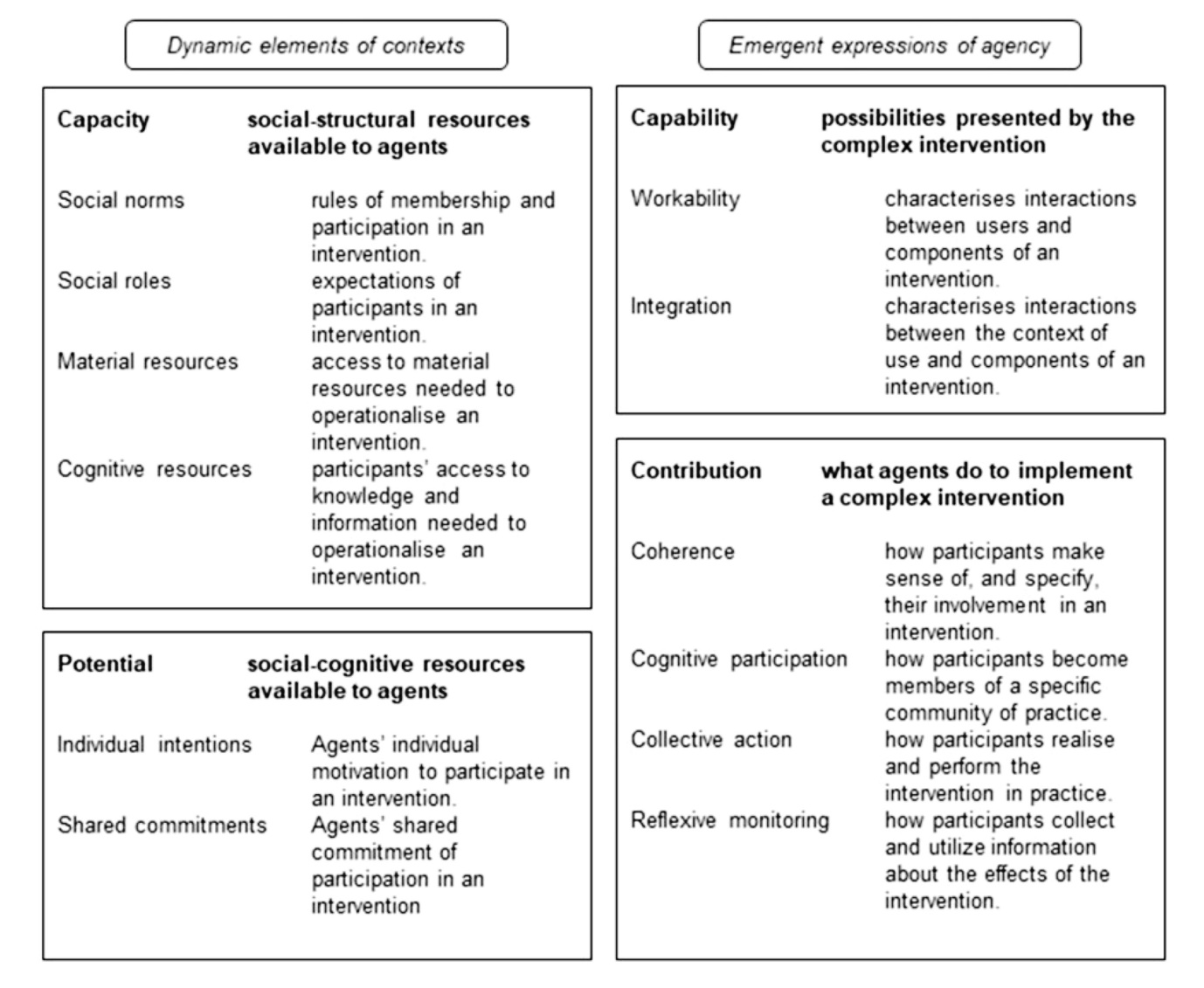


### Potential: staff or other agents’ motivation to implement an intervention

Individual intentions and shared commitments are important to the dynamics of implementation. Individual motivation is necessary for action, especially if agents have a high degree of professional autonomy or personal discretion in their role. However, implementation processes most often depend on collective commitment and collaboration. Organisational members collectively need to value the changes targeted by an intervention and feel confident that the group as a whole can make the necessary changes (C. May 2013). There are two key dimensions:

- *Individual intentions*: agents’ readiness to transform personal beliefs and attitudes into intervention behaviours, affecting agents’ motivation to participate in an intervention.
- *Shared commitments*: agents’ readiness to translate shared beliefs and attitudes into intervention behaviours, affecting agents’ shared commitment to participate in an intervention.

The implication is that an intervention will be more likely to be implemented if staff or other agents both individually and collectively commit to operationalising it in practice. May (2013a) expresses this as a single proposition:

The translation of capacity into collective action depends on agents’ potential to enact the complex intervention. (May, 2013a, p.8)

### Capability: the extent an intervention can be made to fit into agents’ everyday practice

New or modified ways of working (‘ensembles of practice’) are often intended to change people’s expertise and actions. Studies have also shown that the attributes of intervention components themselves affect their use: for example, their physical properties, the assumptions about use and users embedded within them, and the social relations they require for use (C. May 2013). These elements combine to affect how agents interact with an intervention to make it operationally workable, to allocate labour, and to integrate it into practice and the social system. There are two key dimensions:

- *Workability*: how agents allocate work and interact with one another to operationalise an intervention.
- *Integration*: the work carried out to integrate the intervention into existing practices, policies and systems, including procedures to develop accountability and fidelity/consistency of use and resources allocated to its operation.

The implication is that an intervention will be more likely to be sustained if its elements and its associated ways of working can be made operationally workable by staff or other agents, and work is carried out to integrate the intervention into existing policies and procedures. May (2013a) expresses this proposition as follows:

The capability of agents to operationalize a complex intervention depends on its workability and integration within a social system. (May, 2013a, p.5)

### Contribution: the actions carried out by agents to implement an intervention

Contribution is the collective actions of agents to operationalise an intervention. This domain is also known as NPT and was the first of the four domains to be developed (C. R. May et al. 2009). When agents operationalise a complex intervention, they are collectively involved in four social processes (C. May 2013):

- *Coherence or sense-making:* how agents attribute meaning to intervention component, how they make sense of their use and worth and differentiate it from other processes in their social field.
- *Cognitive participation:* agents initiate work that establishes the legitimacy of an intervention and enrols themselves and others (that is, develops ‘buy-in’) into an implementation process. Cognitive participation frames how participants become members of a specific community of practice.
- *Collective action:* agents operationalise the work and distribute and mobilise skills and resources to enact it. Collective action frames how participants realise and perform the intervention in practice.
- *Reflexive monitoring* – agents appraise, formally and informally, the effects and operation of the intervention (May & Finch, 2009, p. 542-546). This dimension frames how participants collect and utilise information about the effects of the intervention.

The implication is that an intervention will have more potential to be implemented if agents invest work in operationalising it in practice. May (2013a) expresses this as the following proposition:

The implementation of a complex intervention depends on agents’ continuous contributions that carry forward in time and space. (May, 2013a, p.9)

### Capacity: the influence of the social context on intervention implementation

Social networks form relational pathways through which different kinds of work are done (C. May 2013). The structure of these social networks affects how information flows between agents (and groups of agents) and affects how work relating to an intervention is communicated and operationalised. Implementation may be required across expansive, physically distributed, social fields (for example, large-scale policy implementation across a health-care system) or may be carried out across much smaller, tightly-knit fields (for example, a team within an organisation). Nonetheless, agents act with a shared set of understandings about the purpose of the network, its relationships and who has power, and rules. Collective action takes place within this social field and this domain frames the basic conditions for people’s expression of agency to invest in implementation (C. May 2013).

There are four key dimensions of capacity:

- *Social norms:* collective rules or understandings of acceptable behaviour that govern agents’ behaviour, rewards and involvement in an implementation process.
- *Social roles:* socially patterned identities within a setting which define expectations of different agents and what they are authorised (or not) to do to operationalise an intervention.
- *Cognitive/informational resources*: whether and how knowledge, information or evidence are disseminated and distributed to agents in an implementation process
- *Material resources*: funding, equipment, physical space, and any other material resources that are mobilised by agents in an implementation process.

The implication is that an intervention will be more likely to be implemented if it aligns with existing norms and roles and there are sufficient material and cognitive resources to support agents to implement an intervention. May (2013a) expresses this as a single proposition:

The incorporation of a complex intervention within a social system depends on agents’ capacity to cooperate and coordinate their actions. (May, 2013a, p.6)

### How the GTI informed data collection and analysis

The GTI informed the research questions examining staff motivation and schools’ capacities to sustain the intervention. The theory was used in the development of the data collection tools: the staff interview guide (assessing each intervention component’s impact on work, attitudes, skills/knowledge and relationships) and the student interview guide (assessing students’ views on the integration of the intervention).

GTI contributed to the analysis of the data by informing the overarching structure of higher-order themes and sub-themes that were developed, enabling the analysis to move beyond merely identifying specific facilitators and barriers, and instead developing explanations that look at broader sustainability processes within schools. The ‘capability’ domain did not inform our final themes; we found that data that could have been coded under ‘capability’ fit sufficiently well under other domains. For example, the ‘workability’ of RP and action groups was related to timetabling and the paucity of time.

To make the analysis process more ‘workable’ for the research questions in the study, while staying close to the language of the theory, the overarching domains were translated: the word “agents” was replaced with “school staff”; “implementation” or “implementation processes” were replaced with “implementation over time” or “implementation processes over time”; and “intervention” was replaced with “Learning Together”. For example, “Social norms: collective rules or understandings of acceptable behaviour that govern agents’ behaviour, rewards and involvement in an implementation process,” was replaced with, “Social norms: collective rules or understandings of acceptable behaviour that govern school staff’s behaviour, rewards and involvement in an implementation process over time.” The descriptions of the dimensions under ‘Potential’ were also slightly re-worded for clarity: individual intentions were described as *individual differences between* school staff in their readiness to transform personal beliefs and attitudes *about how to improve students’ health, wellbeing and behaviour* into intervention behaviours, and shared commitments were described as school staff’s *collective* readiness to translate shared beliefs and attitudes *about how to improve students’ health, wellbeing and behaviour* into intervention behaviours.

**Potential: school staff’s motivation to implement Learning Together over time**

- *Individual intentions*: individual differences in school staff’s readiness to transform personal beliefs and attitudes about how to improve students’ health, wellbeing and behaviour into behaviours prescribed by Learning Together, affecting their motivation to participate in an intervention over time.
- *Shared commitments*: school staff’s collective readiness to translate shared beliefs and attitudes about how to improve students’ health, wellbeing and behaviour into the behaviours prescribed by Learning Together, affecting their shared commitment to participate in the intervention over time.

**Capability: the extent Learning Together could be made to fit into school staff’s everyday practice**

- *Workability*: how school staff allocate work and interact with one another to operationalise Learning Together.
- *Integration*: the work carried out to integrate the Learning Together into existing practices, policies and systems, including procedures to develop accountability and fidelity/consistency of use and resources allocated to its operation over time.

**Contribution: the actions carried out by school staff to implement Learning Together over time**

- *Coherence or sense-making:* how school staff attribute meaning to Learning Together components, how they make sense of their use/ worth and differentiate them from other processes in their work at school.
- *Cognitive participation:* school staff initiate work that establishes the legitimacy of Learning Together and enrols them and others (that is, develops ‘buy-in’) into the implementation process over time.
- *Collective action:* how school staff operationalise Learning Together’s components and distribute and mobilise skills and resources to enact them over time.
- *Reflexive monitoring* – school staff appraise, formally and informally, the effects and operation of Learning Together.

**Capacity: the influence of the social context on Learning Together’s implementation over time**

- *Social norms:* collective rules or understandings of acceptable behaviour that govern school staff’s behaviour, rewards and involvement in the implementation process over time.
- *Social roles:* socially patterned identities within schools which define expectations of different staff and what they are authorised (or not) to do to operationalise Learning Together over time.
- *Cognitive/informational resources*: whether/how knowledge, information or evidence about Learning Together are disseminated and distributed to staff over time.
- *Material resources*: funding, equipment, physical space, and any other material resources that are mobilised by staff to implement Learning Together over time.

### References

May, C., 2013a. Agency and implementation: Understanding the embedding of healthcare innovations in practice. Social Science & Medicine 78, 26–33. <https://doi.org/10.1016/j.socscimed.2012.11.021>

May, C., 2013b. Towards a general theory of implementation. Implementation Science 8. <https://doi.org/10.1186/1748-5908-8-18>

May, C., Finch, T., 2009. Implementing, Embedding, and Integrating Practices: An Outline of Normalization Process Theory. Sociology 43, 535–554. <https://doi.org/10.1177/0038038509103208>
