# Supplementary material for: Staff motivation and schools' capacities to sustain an intervention to prevent bullying and promote wellbeing in English secondary schools: a qualitative study
Source: Front Public Health. 2025 Apr 23;13:1559954. doi: 10.3389/fpubh.2025.1559954 (PMC12057643; doi:10.3389/fpubh.2025.1559954)
Supplement: Supplementary file 3 [file Supplementary_file_3.docx]

# Supplementary file 3: Fidelity scores for Learning Together’s implementation in each school during the trial (years 1 – 3)

|  | **Downton Park** | **Franklyn** | **Fern Grove** | **Bletchford** | **Greenthorne** |
| --- | --- | --- | --- | --- | --- |
| **Years 1 – 2** |  |  |  |  |  |
| Action groups |  |  |  |  |  |
| *Minutes/diaries indicated minimum of six meetings in years 1 and 2* | 1 | 1 | 1 | 1 | 0 |
| *Minutes/diaries indicated review of policies/rules in year 1 or 2* | 0 | 1 | 1 | 0^1^ | 0 |
| *Minutes/diaries indicated implementation of locally decided actions in years 1 and 2* | 0 | 1 | 0 | 1 | 1 |
| *Survey of members indicated good range of students and staff members* | 1 | 1 | 1 | 1 | 1 |
| *Survey of members indicated well led* | 1 | 1 | 1 | 1 | 1 |
| Curriculum |  |  |  |  |  |
| *Survey or interviews indicated five hours/> 1 unit delivered in years 1 and 2* | 1 | 0 | 1 | 0 | 1 |
| Restorative practice |  |  |  |  |  |
| *Attendance logs indicated at least five staff received in-depth training* | 1^2^ | 1 | 1 | 1 | 1 |
| *Staff survey indicated at least 85% of staff report that if there is trouble at this school, staff respond by talking to those involved to help them get on better* | 1 | 1 | 0 | 0 | 0 |
| **Overall score/8**  **Years 1 – 2** | **6** | **7** | **6** | **5** | **5** |
| **Year 3** |  |  |  |  |  |
| Action groups |  |  |  |  |  |
| *Interviews indicated minimum of six meetings in year 3* | 1 | 1 | 0 | 0 | 0 |
| *Interviews indicated implementation of locally decided actions in year 3* | 1 | 1 | 1 | 1 | 0 |
| Curriculum |  |  |  |  |  |
| *Survey or interviews indicated five hours/> 1 unit delivered in year 3* | 1 | 1 | 0 | 0 | 0 |
| Restorative practice |  |  |  |  |  |
| *Staff survey indicated at least 85% of staff report that if there is trouble at this school, staff respond by talking to those involved to help them get on better* | 1 | 1 | 1 | 1 | 1 |
| **Overall score/4**  **Year 3** | **4** | **4** | **2** | **2** | **1** |

^1^This score differs from data in Bonell et al. 2019 but is consistent with trial process evaluation data. ^2^Ibid.
