# Supplementary material for: Staff motivation and schools' capacities to sustain an intervention to prevent bullying and promote wellbeing in English secondary schools: a qualitative study
Source: Front Public Health. 2025 Apr 23;13:1559954. doi: 10.3389/fpubh.2025.1559954 (PMC12057643; doi:10.3389/fpubh.2025.1559954)
Supplement: Supplementary file 4 [file Supplementary_file_4.docx]

# Supplementary file 4: Interview guides for interviews conducted in the first year post-trial

## School staff interview guide

| NOTES  *Previously interviewed?*  *Facilitator was…* | |
| --- | --- |
| INTRODUCTION  The interview should take about 30-40 minutes. We will ask you questions about your views on the sustainability of the Learning Together intervention. Everything we talk about will be completely **confidential.** You will not be identified at any point nor will your school. Also, if you don’t want to answer a particular question, you don’t have to and if you feel uncomfortable or find it difficult to talk about things we can stop the interview at any point.  **Do you agree to take part? We need you to fill in and sign a consent form. Is that OK?** Have you got any questions before we start? | |
| BACKGROUND INFORMATION  **What is your role at the school now?**  **(If not clear), are you a member of the SLT?**  **Do you have teaching responsibilities?**  **How long have you been at the school?** |  |
| ACTIVITIES IN YEAR 3 AND 4   1. **Last year, we spoke to you about how LT had developed within the school.**   **Does that seem right to you?**   1. **Did you go on the in-depth restorative practice training?** 2. **Were you an action group member from the beginning?** | *[insert synopsis]* |
| RESTORATIVE PRACTICE   1. **Has the school continued to use restorative practice this school year?** | *Can you tell me about what has happened?*  **If YES:**   1. How has the school used RP this year? 2. What led the school to continue using RP?   *Prompt on whether views shared by other staff members and SLT*   1. Has anything helped the school to carry on with RP? 2. Have there been any barriers to using RP? 3. How do you know whether RP is being used? (i.e. data collection?)   **If NO:**   1. What do you think lead the school to move away from RP?   *Prompt on whether views shared by other staff members and SLT*   1. What would you have changed about how RP was implemented? 2. What discipline approaches are the school currently using? |
| 1. [If applicable] **Did you gain anything as a teacher from using *restorative practice* in your work?** 2. **Were there any negative impacts on your work from using the *approach*?** | Impact on attitudes?  Impact on skills/knowledge?  Impact on relationships? |
| ACTION GROUPS   1. **Have there been any action groups (or similar groups) this year?** | **If YES**:   1. When? Who attended? New staff/student members? 2. What happened in the group? What is the aim of the group(s) this year? 3. What led the school to continue the groups?   *Prompt on whether views shared by other staff members and SLT*   1. Has anything helped the groups to continue? 2. Have there been any barriers to continuing the groups?   **If an adapted form of the AG group held:**   1. What factors lead to the school moving away from the AGs to group X? 2. Who attended group X? What happened at group X? 3. How have other staff responded to group X? 4. How similar is group X to the AGs?   **If NO:**   1. What factors lead to the school stopping the AGs?   *Prompt on whether views shared by other staff members and SLT*   1. What would you have changed about the AGs? 2. Are there other forums for student voice currently in the school? 3. How did the AGs fit with the other forums for student voice? |
| 1. [If applicable] **Did you gain anything as a teacher from taking part in the action groups?** 2. **Were there any negative impacts on your work from being part of the action groups?** | Impact on attitudes?  Impact on skills/knowledge?  Impact on relationships? |
| CURRICULUM   1. **Were the any of the curriculum materials used by the school last year or this year?** | **If YES:**   1. How are the materials currently being used? 2. What led the school to continue using the materials?   **If NO:**   1. When was the last time that the curriculum materials were used? (e.g. what year, and when - in tutorials or lesson time?) 2. What would you have changed about the curriculum materials? 3. Why do you think the materials stopped being used? |
| 1. [If applicable] **Were the social and emotional learning curriculum materials useful for your work?** | Impact on attitudes?  Impact on skills/knowledge?  Impact on relationships? |
| OTHER INTERVENTIONS AT THE SCHOOL   1. **In the last five years, has the school been involved in any other intervention projects?** 2. [If applicable] **What do you think has helped Project X to continue after the funding stopped?** 3. [If applicable] **What do you think stopped Project Y from continuing after the funding ended?** | *Ask for details on their purpose and activities, when they started, whether continuing to receive external funding, and whether still continuing.* |
| CLOSURE   1. **Are there any other staff members at the school you would suggest I speak to about the sustainability of Learning Together?** 2. [If applicable] **Would you be willing to speak to me again in the summer term to tell me how the action groups/restorative practice have been going this year?** |  |
| 1. **Is there anything else you would like to tell me about your experiences of the intervention that you think is important, and we haven’t covered already?** |  |

## Student interview guide

| NOTES | |
| --- | --- |
| INTRODUCTION  The interview should take about 30 minutes. We will ask you questions about your views on the student-staff groups you took part in and the group you are involved with now. Everything we talk about will be completely **confidential.** You will not be identified at any point nor will your school. Also, if you don’t want to answer a particular question, you don’t have to and if you feel uncomfortable or find it difficult to talk about things we can stop the interview at any point.  **Do you agree to take part? We need you to fill in and sign a consent form. Is that OK?** Have you got any questions before we start? | |
| BACKGROUND INFORMATION   1. **What year are you in?** 2. **Have you been at the school since year 7?** 3. **Are you missing any lessons to speak to me today?** | *If not, when did they join?* |
| ACTION GROUP   1. **How did you become involved in the action group?** 2. **Were any of your friends on the group?** 3. **Who else was in the group?** | *When did you join? (AG from the beginning?)*  *Why did you join?*  *Was there a mix of students in the group in terms of:*  *Year groups?*  *Family background?*  *Ability at school?* |
| **LAST SCHOOL YEAR (2016/17)**   1. **How often did you meet up?** 2. **What normally happened in a meeting?** 3. **Can you give me an example of something in your school that has happened as a result of the group?** 4. **How did the group link with the school community committee/council?**   **THIS SCHOOL YEAR (2017/18)**   1. **Has the group continued this school year?** 2. **How often have you met?** 3. **Have there been any changes in the way the meeting has been run in comparison to last year?** 4. **Can you give me an example of any actions that have happened at the school** this year **as a result of the group?** 5. **What do you enjoy about the group?** 6. **What could be improved?** 7. **Do other people at school know about the group?**      1. **Has being part of the group changed your view of your school?**   **CLOSURE AND THANKS!** | *Describe – Aim? Who involved? What happened? When did it happen?*  *Events?*  *Changes to school rules?*  *Changes to policies?*  *Other?*  *Has anything helped the groups to continue?*  *Have there been any difficulties in continuing the groups?*  *Describe – Aim? Who involved? What happened? When did it happen?*  *Events?*  *Changes to school rules?*  *Changes to policies?*  *Other?*  *Response from teachers?*  *Response from students?* |
