# Supplementary material for: Staff motivation and schools' capacities to sustain an intervention to prevent bullying and promote wellbeing in English secondary schools: a qualitative study
Source: Front Public Health. 2025 Apr 23;13:1559954. doi: 10.3389/fpubh.2025.1559954 (PMC12057643; doi:10.3389/fpubh.2025.1559954)
Supplement: Supplementary file 5 [file Supplementary_file_5.docx]

### Supplementary file 5: Reflexivity statement

At the time of the study, LH and CB identified as White British, middle-class parents, LH a woman in her late thirties, and CB a man in his fifties, living in or close to London, and our characteristics resembled most of the practitioners that were interviewed. We were sympathetic to the difficult, emotional work involved for teachers in managing students’ behaviour, and we assumed that an aspect of the intervention or intervention process was at fault when it was not sustained.

CB was the co-director of the trial and PhD supervisor of LH. CB believed in the value of RP, having been involved in the design of the intervention and its pilot evaluation. LH had been involved in delivering RP in the criminal justice setting as a youth offending worker and knew that the conferences took time organise and needed preparation with young people to be beneficial. Staff experiences in conducting RP met our expectations about its value and work that was involved in carrying it out successfully. We had an interest in the sustainment of RP in the schools.

LH felt more neutral about the likely sustainment of the other components, CB had been enthusiastic about the action groups’ potential to encourage schools to be more inclusive. It was satisfying to discover that several staff reported that the groups helped them to realise the importance of student voice in a meaningful way, even though the original form of the groups was not sustained. We could see that the intervention would have a lasting impact for some staff that was not easily measurable.

For LH, it was hard to qualify the impact of examining an intervention which her supervisor played a key role in designing and implementing. She did not feel any pressure to regard the intervention or its implementation in a positive or negative light; we were both open in our criticism of the features of intervention and its implementation that could have been designed/carried out to promote sustainment. CB’s close knowledge of the intervention helped in the refinement of theory on the particular elements of the intervention and the school context that might affect sustainment.
