# Supplementary material for: Staff motivation and schools' capacities to sustain an intervention to prevent bullying and promote wellbeing in English secondary schools: a qualitative study
Source: Front Public Health. 2025 Apr 23;13:1559954. doi: 10.3389/fpubh.2025.1559954 (PMC12057643; doi:10.3389/fpubh.2025.1559954)
Supplement: Supplementary file 6 [file Supplementary_file_6.docx]

# Supplementary file 6: A summary of staff and students’ reports of Learning Together’s implementation (years 1 to 3) and sustainment (years 4 to 5) by school

## Downton Park

| **Component** | **Years 1 and 2 of the trial** | **Year 3, last year of trial** | **Year 4, 1-year post-trial** | **Year 5, 2-years post-trial** |
| --- | --- | --- | --- | --- |
| *RP* | - All-staff training in RP awareness was delivered. At least six staff received in-depth training on RP. One of these staff delivered RP training to staff in year 2 and it was used as the basis of student mentor training for staff. - RP was integrated into the tutorial time curriculum. - RP principles were presented to year 9 students in PSHE. - RP was used as the basis of peer mentor training for sixth form students. - RP was disseminated to students through posters and assemblies. | - RP used in staff student mentor training and student peer mentor training, newly qualified and support teachers received RP training. - RP used in tutorial time - RP principles taught in one PSHE lesson for Year 9 students. - RP principles discussed in assemblies. - Staff trained in-depth continued to use RP. | - Newly qualified and support teachers continued to be trained in RP. - Staff uncertain whether RP used in tutorial time. - RP principles taught in one PSHE lesson for Year 9 students. - RP principles discussed in assemblies. - Staff trained in-depth continued to use RP. | - Staff trained in-depth continued to use RP. |
| *Action groups and actions* | - At least six action group meetings were held each year during lunchtimes. - There was a good range of students and staff members, students were from multiple year groups. There was consistent staff attendance but inconsistent student attendance. - The action groups were primarily used as a forum for deciding actions to disseminate RP principles and techniques. | - Action groups continued to be held. | - Action groups discontinued. | - A new action group was created that aligned with LT’s ToC but was likely to have occurred without the intervention. |
| *Curriculum* | - At least five hours of more than one unit were delivered in years 1 and 2 in tutorial time. | - Curriculum delivered in tutorial time (integrated with RP). | - Staff uncertain whether curriculum continued to be used in tutorial time. | - Curriculum discontinued. |

## Franklyn

| *RP* | - All-staff training in RP awareness was delivered. At least six staff received in-depth training on RP, and one staff member communicated the principles of RP at a staff INSET day. | - RP used in discipline process in *Red* meetings. - Staff trained in-depth continued to use RP. | - RP used in discipline process in *Red* meetings. - Staff trained in-depth continued to use RP. | - RP continued to be used in discipline process in *Red* meetings. - Staff trained in-depth continued to use RP. - Staff received external training in student resilience that had embodied the principles of RP. |
| --- | --- | --- | --- | --- |
| *Action groups and actions* | - At least six action group meetings were held each year in lesson time. - There was a good range of students and staff members, students were from multiple year groups. There was consistent student attendance but inconsistent staff attendance. The group was led by a different staff member in years 1 and 2. - The group carried out a review of school policies/rules to reduce risk behaviours and promote wellbeing. They produced the ‘Franklyn pyramid’ which explained the escalating stages of sanctions. - Locally decided actions were implemented. Franklyn introduced award assemblies for each year group every half term. It introduced a more severe sanction for late attendance, and ‘*Red’* meetings – a restorative meeting between a teacher and student if poor behaviour continued. | - Action groups discontinued. - Two new action groups created focused on social inequalities that aligned with LT’s ToC and may have occurred in part because of the intervention. - The Franklyn pyramid and locally decided actions continued to be implemented. | - New action groups continued, reviewed school policies, organised events and influenced student and staff training. - The Franklyn pyramid and locally decided actions continued to be implemented. | - New action groups continued, organised events and influenced student and staff training. - The Franklyn pyramid and locally decided actions continued to be implemented. |
| *Curriculum* | - Partially delivered the curriculum in years 1 and 2 in PSHE classes but not to the required fidelity level. | - Curriculum delivered in tutorial time. | - Curriculum discontinued. | - Curriculum discontinued |

## Fern Grove

| *RP* | - All-staff training in RP awareness was delivered. At least six staff received in-depth training on RP, and one staff member held a further RP training session for other staff members in year 2. - One senior staff member disseminated RP principles through observation and feedback. | - Staff trained in-depth continued to use RP. | - Staff trained in-depth continued to use RP. | - Staff trained in-depth continued to use RP. |
| --- | --- | --- | --- | --- |
| *Action groups and actions* | - At least six action group meetings were held each year in lesson time. - There was a good range of students and staff members, students were from multiple year groups. There was consistent student attendance but inconsistent staff attendance. The group was led by a different staff member in years 1 and 2. - The group carried out a review of school policies/rules to reduce risk behaviours and promote wellbeing. They created a list of behavioural expectations – “rules of conduct” (ROC). Students went on ‘learning walks’ to observe teachers’ use of the behaviour policy. | - Action group continued. - Fern Grove’s ROC continued to be used. | - Action group discontinued. - Three new action groups created focused on teaching and learning, behaviour and wellbeing, and student voice, two of which aligned with LT’s ToC and were considered an evolution of the intervention. - A new student voice coordinator was appointed. - Fern Grove’s ROC continued to be used. | - New action groups discontinued. - Student voice coordinator remained in post. - Fern Grove’s ROC continued to be used. |
| *Curriculum* | - At least five hours of more than one unit were delivered in years 1 and 2. It was delivered through drama lessons in year 1 and in tutorial time in year 2. | - Curriculum discontinued. | - Curriculum discontinued. | - Curriculum discontinued. |

## Bletchford

| *RP* | - All-staff training in RP awareness was delivered. At least six staff received in-depth training on RP. One of these staff delivered further RP training to staff in year 2. | - RP training rolled-out across the school. - Staff trained in-depth continued to use RP. | - RP integrated into the school as part of a new discipline system called Education for All. Staff trained in using the new system. - Staff trained by external or internal trainers used RP. | - RP integrated into the school as part of Education for All. Staff trained in using the new system. - Staff trained by external or internal trainers used RP. |
| --- | --- | --- | --- | --- |
| *Action groups and actions* | - At least six action group meetings were held each year during lunchtimes. - There was a good range of students and staff members, students were from multiple year groups. However, student attendance was inconsistent and there was high staff turnover. - Locally decided actions were implemented. Bletchford set up a peer mentoring programme (which ended in year 2). - The action groups were primarily used as a forum for deciding actions to disseminate RP principles and techniques. | - Action group discontinued. | - Action group discontinued. | - Action group discontinued. |
| *Curriculum* | - Partially delivered the curriculum, in PSHE classes in year 1 and then some of the materials were used to establish student training in RP principles in tutorial time in year 2 (but not to the required fidelity level). | - Curriculum discontinued. | - Curriculum discontinued. | - Curriculum discontinued. |

## Greenthorne

| *RP* | - All-staff training in RP awareness was delivered. At least five staff received in-depth training on RP, and one staff member held a further RP training session for other staff members in year 1. - One staff member created a summary document of the RP approach and prompts to use and sent it out to staff. - One staff member created a RP worksheet for students to complete in detentions. | - Staff trained in-depth continued to use RP. | - Staff trained in-depth continued to use RP. - RP was written into discipline procedures. | - Staff trained in-depth continued to use RP. - RP remained written into discipline procedures. |
| --- | --- | --- | --- | --- |
| *Action groups and actions* | - The action group met a few times each year in lesson time. - There was a good range of students and staff members, but students were from one year-group only (year 8 in year 1, moving on to year 9 in year 2). The group was led by a different staff member in years 1 and 2. - Locally decided actions were implemented. A pastoral room was made available to students at lunchtime, however, a senior leader in year 4 said although they had let the group ‘claim’ it, it had already been planned and was not a result of the intervention. | - Action groups discontinued. | - Action group discontinued. | - New action group created that aligned with LT’s ToC and may have occurred because of the intervention. |
| *Curriculum* | - At least five hours of more than one unit were delivered in one day in year 1, and in PSHE classes in year 2. | - Curriculum discontinued | - Curriculum discontinued. | - Curriculum discontinued. |

LT - Learning Together, TOC - Theory of Change, PSHE – Personal Social Health and Economics, ROC – Rules of conduct
