# Supplementary material for: Staff motivation and schools' capacities to sustain an intervention to prevent bullying and promote wellbeing in English secondary schools: a qualitative study
Source: Front Public Health. 2025 Apr 23;13:1559954. doi: 10.3389/fpubh.2025.1559954 (PMC12057643; doi:10.3389/fpubh.2025.1559954)
Supplement: Supplementary file 7 [file Supplementary_file_7.docx]

Supplementary file 7: Perceived intervention effectiveness

Perceived effectiveness of RP: “I think it’s definitely stopped some major bust ups between kids”

All staff interviewed had been motivated to sustain RP approaches in their own practice as they had appraised it as an effective way of drawing most pupils into conversations about behaviour, improving peer relationships and their own relationships with pupils (see quote in section title), and could improve pupil discipline.

*RP was the starting point to getting staff - including everybody, even myself included – to think about how our...our actions and reactions are actually the central cause of whether it's going to be a positive outcome or a worse outcome.* Joe, SLT, Bletchford, year 4

Staff members were also mindful of whether their parent community was likely to approve RP to manage behaviour over more punitive approaches. Staff positively differentiated RP from the use of detentions because it gave pupils the opportunity to develop empathy, learn from incidents and how to negotiate a positive way forward.

*Encouraging the student to also understand why that's happening. Get them to agree to it, rather than it feeling, you know, like it's imposed. Understand that they do, that something does need to happen.* Callum, staff, Downton Park, year 4

Perceived effectiveness of the action groups: “*We couldn't really have another body… doing exactly the same thing*”

Staff’s reported the effectiveness of the action groups was based on whether the group achieved purposeful actions in its first two years, whether it was perceived to add something valuable or different to existing student voice groups (e.g. student council, see quote in section title), and whether staff found it to be a meaningful experience, giving them insight into their own and pupils’ views about school and the value of listening to pupils.

*They [the action group] had helped us rewrite our behaviour policy, they had had discussions and were really our... student voice group on there. And they were brilliant, because the other existing mechanisms within the school that existed for that were things like the student council, but that was dominated by our best-behaved students who were most engaged*. David, SLT, Fern Grove, year 4.

*The action groups were...the smallest part for us. So they...I don't really feel like the action groups...had any direct impact on what we did as a school.* Jenny, staff, Bletchford, year 4

The groups’ ability to meet these criteria was also constrained by the senior leadership team’s decision whether to allow the group to revise the school’s rules or policies around behaviour and/or attitude to learning, necessary for making the groups’ purpose authentic. Even though the original action groups were positively regarded by staff at Fern Grove and Franklyn, they were not sustained beyond three years, as staff reported that embedding the actions raised by the groups and ensuring actions were consistently delivered required staff training and monitoring that was beyond the group’s remit.

*I think it's just....a lack of consistency. And I think the teachers believe in themselves that...the new structure or the new...plans that...the teachers at the top of the school want to implement, they...aren't very effective.* Craig, student, Franklyn, year 4

*You’ve got more staff now involved in the RED system [student-teaching disciplinary meeting based on RP principles], although at the moment there’s still probably a lack of commonality in terms of how that’s delivered. So I think, because we’ve had lots of new staff come in and we haven’t necessarily trained everybody up in the restorative approach.* Matt, SLT, Franklyn, year 5

Perceived effectiveness of the curriculum: “*The lessons were very, very basic*”:

Learning Together’s curriculum was considered ineffective because the resources were considered inferior to schools’ existing PSHE provision. Staff and the external facilitator reported that Learning Together’s curriculum added minimal value to Bletchford’s existing high quality and extensive PSHE provision, and it was not highly regarded by staff at Greenthorne, Franklyn and Fern Grove. Although staff at Downton Park gave positive feedback on their adaption of the curriculum for disseminating RP approaches to students, it was not sustained beyond year 4 when responsibility for its delivery was transferred to staff that were not involved in Learning Together.

*The feedback that I got from the [PSHE] teachers who taught it in year 2 was the lessons were very, very basic,.. and I don't know if they were just supposed to be the bones for a lesson and then we're supposed to put the work in, I think they thought that it would be a fully formed lesson so...... or we already had similar stuff in place that we just continue what we're doing. Colin, SLT, Greenthorne, year 4*
