# Supplementary material for: Staff motivation and schools' capacities to sustain an intervention to prevent bullying and promote wellbeing in English secondary schools: a qualitative study
Source: Front Public Health. 2025 Apr 23;13:1559954. doi: 10.3389/fpubh.2025.1559954 (PMC12057643; doi:10.3389/fpubh.2025.1559954)
Supplement: Supplementary file 8 [file Supplementary_file_8.docx]

Supplementary file 8: how staff described the responsibilities of different staff in the school in relation to behaviour management

| **Level of responsibility** | **Position** | **Responsibility for behaviour** |
| --- | --- | --- |
| *Support and specialist staff* | Auxiliary staff – reception staff, canteen staff, office staff | Modelling behaviour through daily interaction with all students. |
|  | Pastoral support staff, e.g. pastoral support officer. Learning support staff, e.g. teaching assistants. External health staff – health link worker, school nurse, school counsellors | Welfare support for students with additional social, emotional, behavioural, and/or learning needs. |
| *Teaching staff without additional responsibilities* | Cover/supply teachers  Teachers in training and newly qualified teachers  Subject teachers without additional responsibilities | Management of student behaviour in classrooms, corridors, and in school outside areas. |
| *Teaching staff with lower-level responsibilities* | Teachers who are form tutors | Management of student behaviour in classrooms, corridors, and in school outside areas. First point of contact for students with additional pastoral or learning needs in their form class. |
| *Teaching staff with middle management responsibilities* | Assistant Heads of Year/Year Leads | Management of student behaviour in classrooms, corridors, and in school outside areas. Additional pastoral responsibilities for the year group, which can include behaviour and attendance. |
|  | Heads of Year/Year Leads | Management of student behaviour in classrooms, corridors, and in school outside areas. Additional oversight and responsibility for pastoral needs and behaviour in year group. |
|  | Head of Departments | Management of student behaviour in classrooms, corridors, and in school outside areas. Additional oversight and responsibility for behaviour management in subject department. |
|  | Heads of Key Stage (higher middle management) | Management of student behaviour in classrooms, corridors, and in school outside areas. Additional oversight and responsibility for pastoral needs and behaviour across multiple year groups. |
| *Teaching staff with high level management responsibilities* | Assistant head teachers  Associate head teachers  Head teachers | Management of student behaviour in classrooms, corridors, and in school outside areas. Responsibility for cross-year group issues or multiple year groups, such as oversight of pastoral care, key stages, or sixth form. Responsibility for students with serious behaviour problems, including involvement in the decision to exclude a student. |
| *Other groups of influence* | Governors, parents, student bodies, for example, prefects, sixth form leadership, school council. | Consult with school middle and high-level leaders and have influence over welfare and behaviour policies. |
